# Blocking cell cycle progression through CDK4/6 protects against chronic kidney disease

**DOI:** 10.1172/jci.insight.158754

**Published:** 2022-06-22

**Authors:** Yosuke Osaki, Marika Manolopoulou, Alla V. Ivanova, Nicholas Vartanian, Melanie Phillips Mignemi, Justin Kern, Jianchun Chen, Haichun Yang, Agnes B. Fogo, Mingzhi Zhang, Cassianne Robinson-Cohen, Leslie S. Gewin

**Affiliations:** 1 Division of Nephrology and Hypertension, Department of Medicine, Washington University St. Louis, St. Louis, Missouri, USA.; 2 Division of Nephrology and Hypertension, Department of Medicine, and; 3 Department of Pathology, Microbiology, and Immunology, Vanderbilt University Medical Center (VUMC), Nashville, Tennessee, USA.; 4 Department of Medicine, Veterans Affairs Hospital, St. Louis VA, St. Louis, Missouri, USA.

**Keywords:** Nephrology, Cell cycle, Fibrosis

## Abstract

Acute and chronic kidney injuries induce increased cell cycle progression in renal tubules. While increased cell cycle progression promotes repair after acute injury, the role of ongoing tubular cell cycle progression in chronic kidney disease is unknown. Two weeks after initiation of chronic kidney disease, we blocked cell cycle progression at G1/S phase by using an FDA-approved, selective inhibitor of CDK4/6. Blocking CDK4/6 improved renal function and reduced tubular injury and fibrosis in 2 murine models of chronic kidney disease. However, selective deletion of cyclin D1, which complexes with CDK4/6 to promote cell cycle progression, paradoxically increased tubular injury. Expression quantitative trait loci (eQTLs) for *CCND1* (cyclin D1) and the CDK4/6 inhibitor *CDKN2B* were associated with eGFR in genome-wide association studies. Consistent with the preclinical studies, reduced expression of *CDKN2B* correlated with lower eGFR values, and higher levels of *CCND1* correlated with higher eGFR values. CDK4/6 inhibition promoted tubular cell survival, in part, through a STAT3/IL-1β pathway and was dependent upon on its effects on the cell cycle. Our data challenge the paradigm that tubular cell cycle progression is beneficial in the context of chronic kidney injury. Unlike the reparative role of cell cycle progression following acute kidney injury, these data suggest that blocking cell cycle progression by inhibiting CDK4/6, but not cyclin D1, protects against chronic kidney injury.

## Graphical abstract

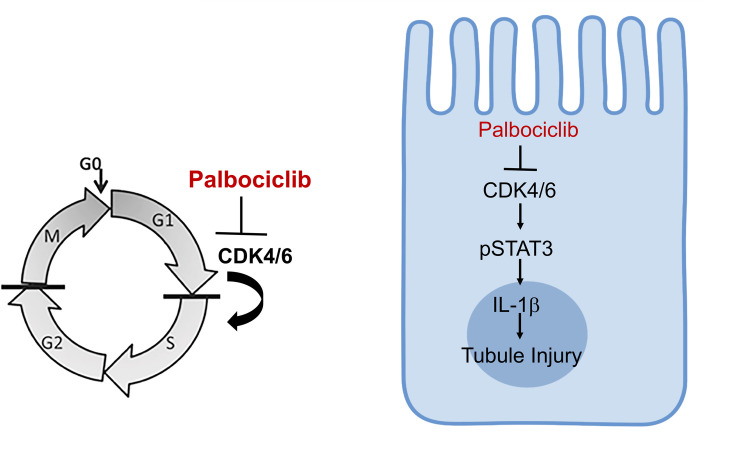

## Introduction

Chronic kidney disease (CKD) affects nearly 15% of the global population and is increasing in prevalence due to the global rise of diabetes and hypertension, the 2 main causes of CKD. Different cell types contribute to CKD progression (e.g., myofibroblasts, macrophages, endothelial cells), but the tubular compartment is a critical target and mediator of kidney injury. Isolated injury to the tubules in murine models is sufficient to cause tubulointerstitial fibrosis, the pathologic hallmark of CKD (1, 2). Among tubular segments, the proximal tubule is particularly vulnerable to acute and chronic kidney injuries, and the injured proximal tubule is considered a potent mediator of CKD progression through production of proinflammatory and profibrotic cytokines (3). Proximal tubules’ response to injury is a critical determinant of whether the kidney undergoes repair or continued tubular injury and fibrosis.

After injury, the most vulnerable tubular cells die through apoptosis or necrosis, while the remaining tubule cells dedifferentiate and reenter the cell cycle. Tubule proliferation following an ischemic or septic event promotes kidney recovery after an acute kidney injury (AKI) (4). However, in the chronically injured kidney, the role of ongoing tubular cell cycle progression is unknown. Our group previously found that selectively deleting the TGF-β type II receptor (TβRII) in proximal tubule cells worsened the response to murine models of CKD, and this was associated with altered cell cycle progression in tubular cells in vitro (5). Consistent with these data, single-cell sequencing showed that human CKD kidneys had more actively cycling proximal tubules compared with kidneys from patients with normal renal function (6). However, these findings do not determine whether the increased cell cycling causes or just reflects ongoing injury. Recent transcriptomic and genome-wide association studies identified dachshund homolog 1 (DACH1), which suppresses tubular cell cycle progression, as having protective effects on CKD (7). Though suggestive that ongoing tubular cell cycling may be detrimental, few studies have directly manipulated cell cycle progression in the chronically injured kidney to determine how tubular cell cycle alters tubular injury and fibrosis.

Most proximal tubules in the uninjured kidney are quiescent (G0) but, upon injury, reenter the cell cycle at G1, which is the first cell cycle checkpoint. Actively cycling cells progress from G1/S phase (DNA synthesis); then to the second checkpoint, G2; and finally to M (mitosis). Work has established that some tubule cells arrest at G2/M following injury, and these G2-arrested cells play an important role in CKD progression through production of profibrotic growth factors like TGF-β and CTGF (8). However, little is known about the role of cell cycle progression at the G1/S transition in chronically injured tubules. Cyclin dependent kinases 4 and 6 (CDK4/6) combine with cyclin D1 to phosphorylate retinoblastoma protein (Rb), leading to the liberation of E2F, a transcription factor that promotes expression of genes related to G1/S phase progression. The FDA-approved drug palbociclib is a highly selective inhibitor of CDK4/6 that is currently used to treat patients with breast cancer and provides an important tool to assess how G1/S cell cycle progression alters CKD progression.

In this manuscript, we use a cell cycle reporter mouse to confirm that cell cycle is persistently altered in the chronically injured proximal tubules. Blocking CDK4/6 in mice after 2 different models of CKD significantly reduced tubular cell cycle progression, ameliorated tubular injury, preserved renal function, and decreased tubulointerstitial fibrosis. Surprisingly, selective tubular deletion of cyclin D1 worsened, rather than improved, tubular injury in murine CKD. Human expression quantitative trait loci (eQTLs) and genome-wide association study (GWAS) data are consistent with the murine studies showing that inhibiting CDK4/6, but not cyclin D1, protects against CKD. Mechanistically, CDK4/6 inhibition reduced proximal tubule cell death through a pathway involving STAT3/IL-1β.

## Results

### Proximal tubules lacking TGF-β receptors have increased cell cycle progression after injury.

**Figure 1:**
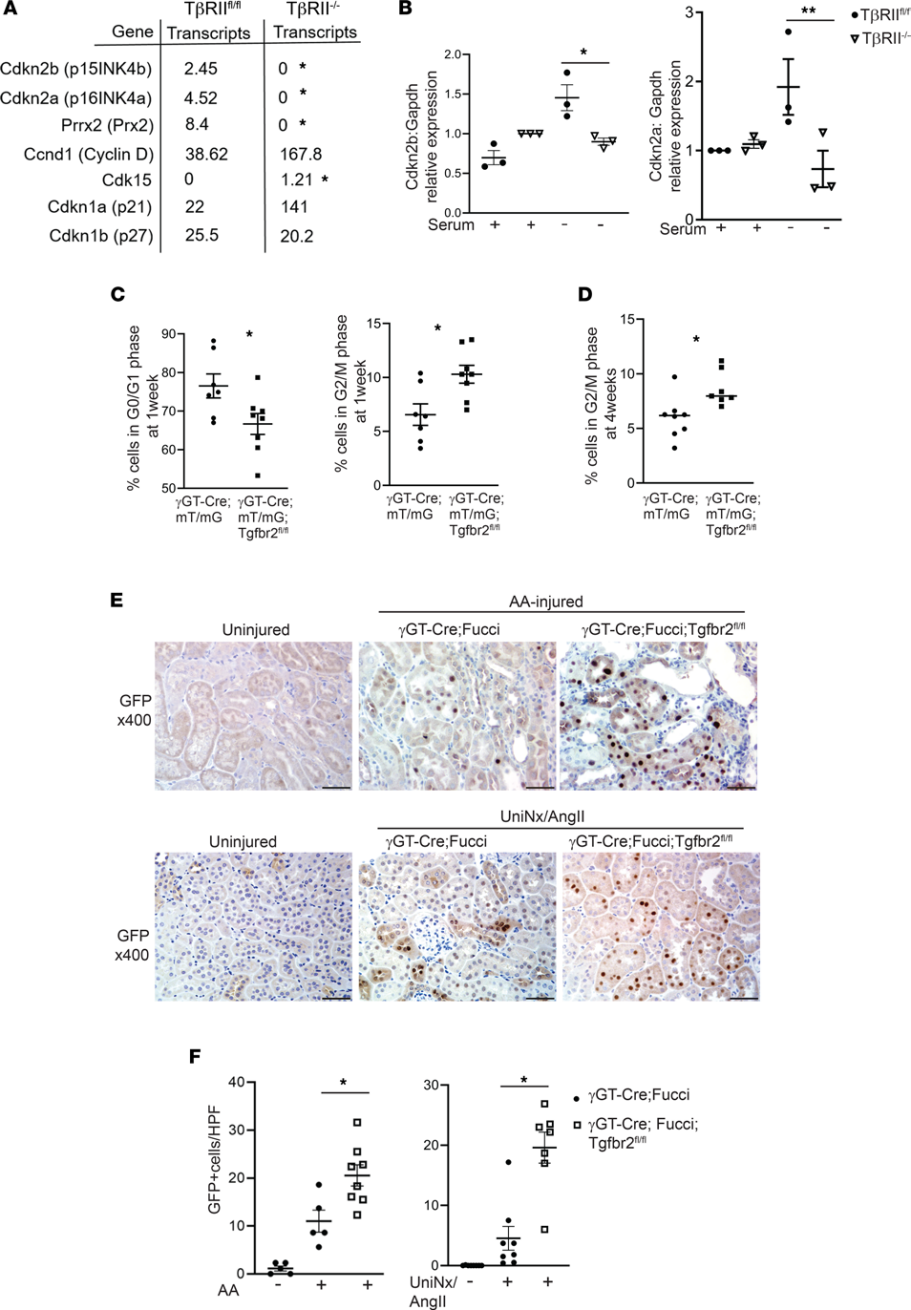
Injured proximal tubules lacking TGF-β receptor have increased cell cycle progression. (**A**) Transcript levels of genes related to cell cycle progression from RNA-Seq on serum-starved innermedullary collecting duct cells ± the TGF-β type II receptor (TβRII). (**B**) Gene expression of Cdkn2b (p15) and Cdkn2a (p16) were measured in conditionally immortalized PT cells using qPCR with Gapdh as a housekeeping gene. (**C** and **D**) Cell cycle of the PT cells (i.e., GFP^+^) from mice with TβRII intact (γGT-Cre;mT/mG) compared with those with TβRII selectively deleted in PT cells (γGT-Cre;mT/mG;Tgfbr2^fl/fl^) at 1 (**C**) or 4 weeks (**D**) after AA injury. (**E** and **F**) Actively cycling cells were detected and quantified using the R26Fucci2aR (Fucci) reporter with IHC using GFP antibody to detect Venus^+^ (S, G2, M) proximal tubule cells in mice with TβRII intact (γGT-Cre;Fucci) and TβRII selectively deleted in the proximal tubule (γGT-Cre;Fucci;Tgfbr2^fl/fl^) in both the AA and UniNx/AngII models. Statistical significance was determined by Benjamini-Hochberg correction for multiple testing in **A** and using 2-tailed Student’s *t* test for others. *P < 0.05 and **P < 0.01. Scale bar: 50 μm. PT, proximal tubule; AA, aristolochic acid; UniNx/AngII, uninephrectomy/angiotensin II).

Our group previously showed that deleting the TβRII unexpectedly worsened the response to chronic kidney injury, suggesting that some TGF-β signaling may be important for epithelial repair (5). To determine the mechanism, we analyzed RNA-Seq data performed on inner medullary collecting duct (IMCD) cells with and without TβRII, as previously characterized (5). Several genes related to cell cycle were significantly altered (Figure 1A). In particular, Cdkn2a (p16) and Cdkn2b (p15) — cell cycle inhibitors that repress CDK4/6-mediated cell cycle progression from G1 to S — were suppressed in TβRII^–/–^ IMCD cells. We then validated that Cdkn2a/b gene expression was significantly reduced in conditionally immortalized proximal tubule (PT) cells with TβRII deleted in vitro and previously characterized (Figure 1B) (9). These data show that deleting TβRII significantly suppressed gene expression of the G1/S cell cycle inhibitors Cdkn2a and Cdkn2b in vitro, and our group previously showed that these TβRII^–/–^ PT cells had increased G2/M arrest after injury in vitro (5).

Cell cycle in vitro may not accurately reflect in vivo responses, so we investigated how TβRII affects PT cell cycle in vivo by crossing γGT-Cre;Tgfbr2^fl/fl^ mice (9) with the mT/mG reporter mouse, which labels Cre-expressing cells with membrane-bound GFP (10). Mice were injured by aristolochic acid (AA), a toxin that targets the PT leading to tubulointerstitial fibrosis and causes human CKD. By gating on GFP^+^ cells, we previously demonstrated that AA leads to persistent PT-specific cell cycle changes 4 weeks after injury (10). Mice lacking TβRII in PT cells (γGT-Cre;mT/mG;Tgfbr2^fl/fl^) had fewer PT cells in G0/G1 and more in G2/M 1 week after AA compared with those with TβRII intact (γGT-Cre;mT/mG) (Figure 1C). The difference in PT-specific G2/M persisted at 4 weeks after AA-induced injury (Figure 1D). We confirmed these findings using a cell cycle reporter mouse that specifically labels PT cells in G0/G1 with mCherry and those in S/G2/M with mVenus (γGT-Cre;R26Fucci2aR, hereafter referred to as “γGT-Cre;Fucci”), as previously published (10, 11). The mice lacking TβRII in PT cells had increased cell cycling (mVenus^+^ cells detected by GFP immunostaining) compared with those with TβRII intact (Figure 1E). To confirm that these findings are not specific to the AA model, we also injured the Fucci mice using uninephrectomy plus angiotensin II infusion (UniNx/AngII), a hypertensive model that induces tubulointerstitial fibrosis (12). Consistent with the AA model, mice lacking PT-specific TβRII had more PT cells actively cycling (i.e., GFP^+^ cells; Figure 1, E and F). Taken together, deleting TβRII significantly reduced Cdkn2a/b expression in vitro and increased PT-specific cell cycling in 2 models of murine CKD.

### Inhibiting CDK4/6 ameliorated the response to chronic kidney injury.

**Figure 2:**
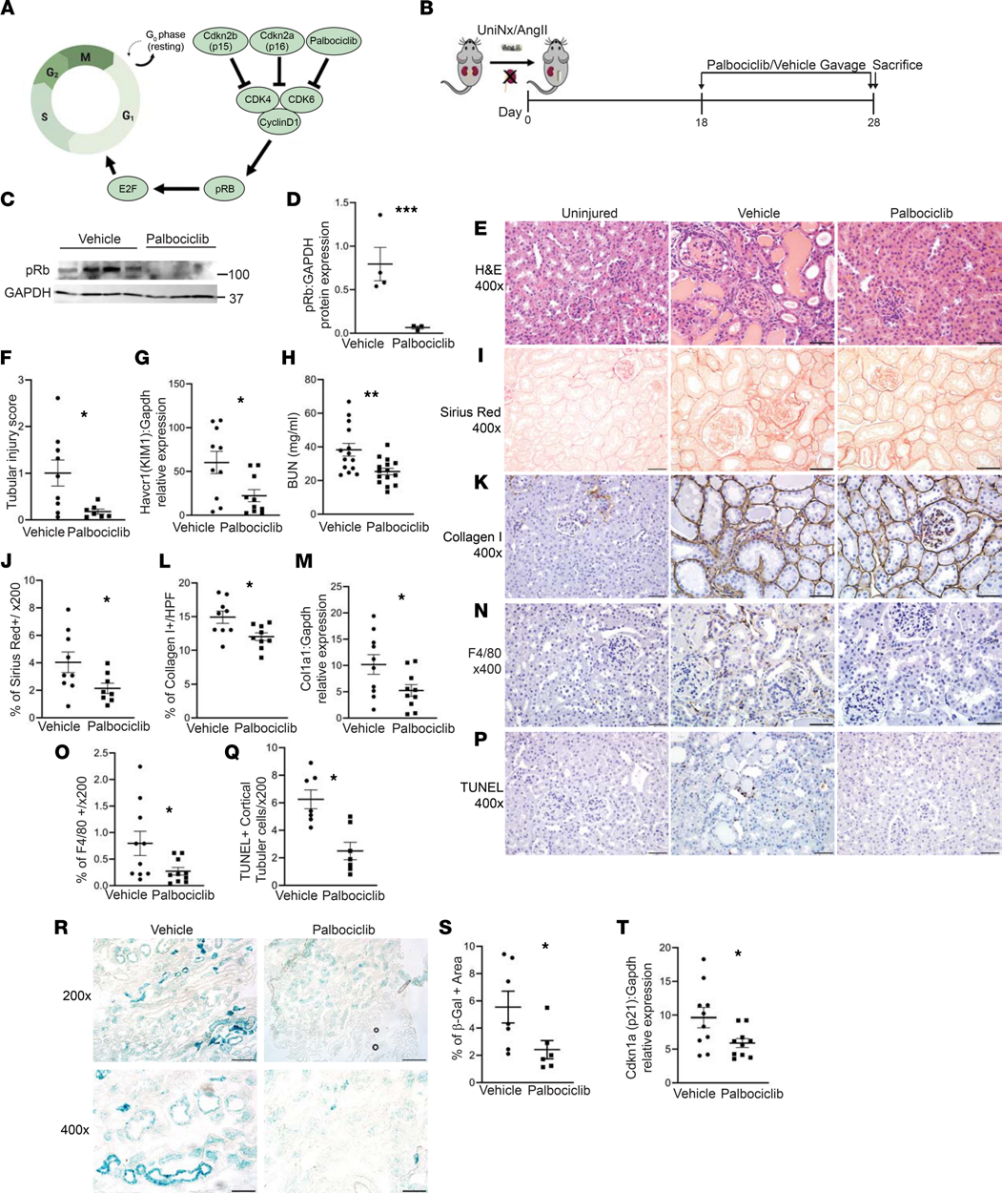
Blocking CDK4/6 reduces tubular injury, fibrosis, and senescence after UniNx/AngII. (**A** and **B**) Diagram of cell cycle progression at G1/S and injury schematic for UniNx/AngII. (**C** and **D**) UniNx/AngII-treated kidney lysates were blotted for retinoblastoma protein (Rb) phosphorylated at serine807/811, the target of CDK4/6. (**E** and **F**) H&E of kidneys injured by UniNx/AngII and treated with either gavage or palbociclib, a CDK4/6 inhibitor (**E**) and quantification of tubular injury (**F**). (**G** and **H**) Gene expression of KIM-1 from injured kidney cortices measured by qPCR and BUN from plasma at the time of sacrifice. (**I**–**L** and **N**–**Q**) Staining and quantification of Picrosirius red (**I** and **J**), collagen I (**K** and **L**), F4/80 (**N** and **O**), and TUNEL (**P** and **Q**). (**M**) Collagen I gene expression (Col1a1) measured from renal cortices using qPCR. (**R**–**T**) Senescence assessed by β-galactosidase staining on frozen sections (**R** and **S**) and gene expression of p21 (Cdkn1a) on injured renal tissue (**T**). The percentage of β-galactosidase^+^ area was quantified on 400× fields (**S**). *P < 0.05,**P < 0.01, and ***P < 0.001 calculated by 2-tailed Student’s *t* test. Scale bar: 50 μm for 400× and 100 μm for 200×.

Conditional deletion of TβRII in PT cells worsened the response to chronic kidney injury (5) and increased PT-specific cell cycling. Although TGF-β signaling has a direct effect on Cdkn2a/b, it is unclear whether the TβRII-dependent alterations in cell cycling in vivo cause the exacerbated injury response or merely reflect increased injury. To determine if there is a mechanistic link between increased cell cycling at G1/S and tubular injury, we administered palbociclib — an FDA-approved specific inhibitor of CDK4/6, the targets of Cdkn2a/b (Figure 2A) — or vehicle to mice 18 days after UniNx/AngII (Figure 2B). We confirmed that there were no differences in blood pressure between palbociclib- and vehicle-treated mice (Supplemental Figure 1; supplemental material available online with this article; https://doi.org/10.1172/jci.insight.158754DS1). CDK4/6 inactivates Rb through inhibitory phosphorylation, and, as expected, palbociclib-treated kidneys had less phosphorylated Rb (Figure 2, C and D). We confirmed that palbociclib reduced cell cycle progression in the UniNx/AngII-treated kidneys using staining for Ki-67, measuring G1/S/G2/M, EdU for S phase, and phosphorylated histone H3 (pHH3) for G2/M (Supplemental Figure 2).

Blocking CDK4/6 reduced UniNx/AngII-induced tubule injury as measured by tubule injury scoring (see Methods) and kidney injury molecule 1 (KIM-1) gene expression in renal cortices (Figure 2, E–G). Renal function, measured by blood urea nitrogen (BUN), was also significantly reduced (Figure 2H). CDK4/6 inhibition also decreased renal fibrosis measured by Picrosirius red staining, collagen I IHC, and gene expression (Figure 2, I–M). Inflammation is an important component of kidney injury, and macrophage expression in human CKD biopsies predicts worse renal survival (13). Macrophage infiltration, measured by F4/80 staining, was reduced in palbociclib-treated UniNx/AngII-injured kidneys (Figure 2, N and O). Tubule cell death contributes to tubular atrophy, a critical component of tubulointerstitial fibrosis; cortical tubule cells that were TUNEL^+^, marking apoptotic and necrotic cells, were significantly reduced in palbociclib-treated mice (Figure 2, P and Q). There are concerns, primarily based on oncology literature, that blocking cell cycle progression from G1/S phase induces tubular senescence, an irreversible state of cell cycle arrest in which cells produce proinflammatory and profibrotic cytokines. We measured senescence-associated β-galactosidase expression in frozen sections and found that palbociclib actually reduced β-galactosidase expression (Figure 2, R and S). Gene expression of p21 (Cdkn1a), another indicator of senescence, was similarly suppressed by CDK4/6 blockade in vivo (Figure 2T).

**Figure 3:**
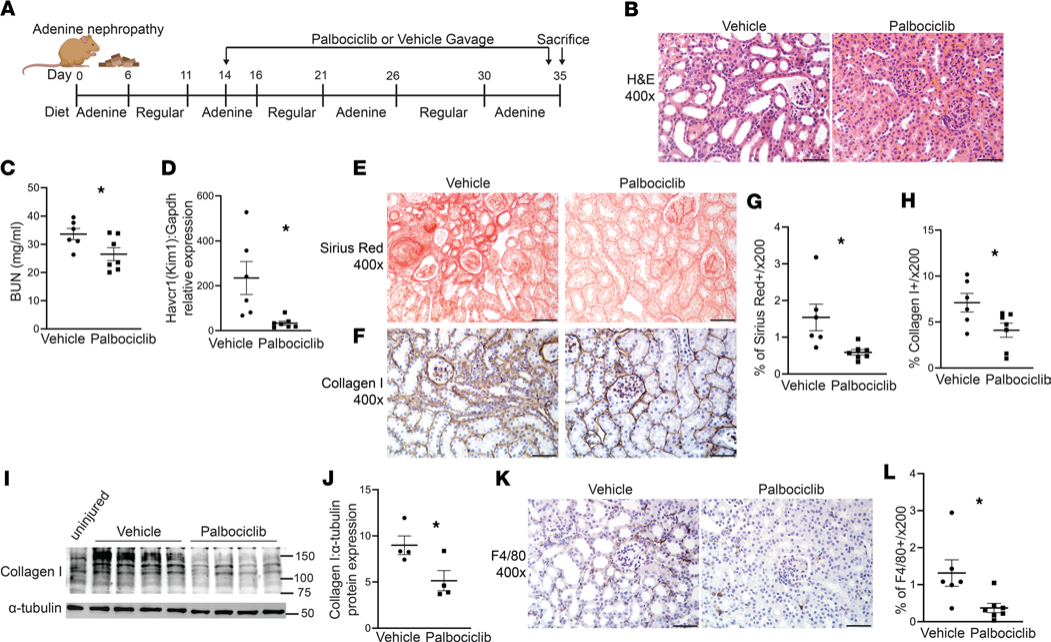
CDK4/6 inhibition ameliorates renal function and fibrosis after adenine nephropathy. (**A**) Schematic for the adenine nephropathy model and palbociclib administration. (**B**–**D**) Following adenine nephropathy, H&E of kidney cortices (**B**), BUN at time of sacrifice (**C**), and KIM-1 (Havcr1) gene expression in renal cortices by qPCR (**D**). (**E**–**J**) Picrosirius red and collagen I staining and quantification (**E**–**H**), and collagen I immunoblots (collagen I MW of 130 kDa) on renal cortical lysates with α-tubulin as loading control and quantified (**I** and **J**). (**K** and **L**) F4/80 staining and quantification in renal cortices. *P < 0.05 calculated by 2-tailed Student’s *t* test, and scale bar: 50 μm.

We confirmed the protective effect of blocking CDK4/6 in the adenine nephropathy model, which induces crystal formation in PT cells, leading to inflammation and tubulointerstitial fibrosis (14). Palbociclib reduced tubule injury, KIM-1 gene expression, and BUN when given 2 weeks after the initiation of adenine nephropathy compared with vehicle-treated mice (Figure 3, A–D). Tubulointerstitial fibrosis and macrophage accumulation were also attenuated by CDK4/6 inhibition, as measured by Picrosirius red, collagen I IHC and immunoblots, and F4/80 IHC (Figure 3, E–L). Thus, blocking CDK4/6 after the initiation of 2 different CKD murine models improved tubular injury, renal function, and tubulointerstitial fibrosis.

### Deletion of tubular cyclin D1 worsens response to chronic kidney injury.

**Figure 4:**
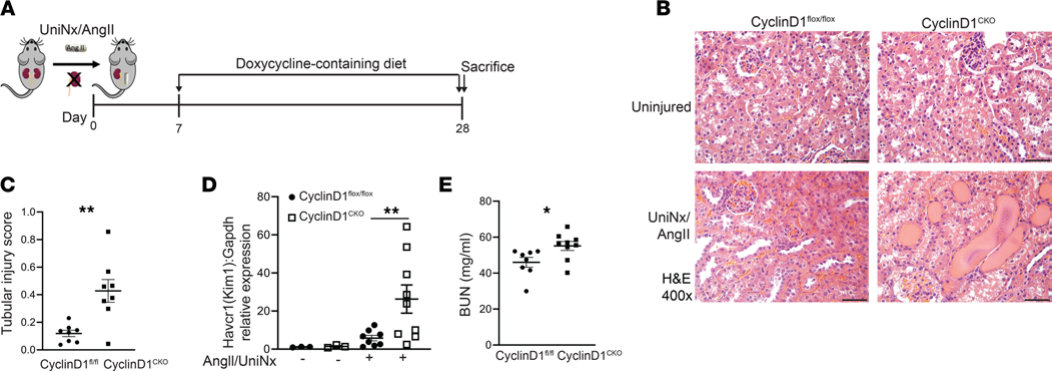
Selective tubular deletion of cyclin D1 exacerbates UniNx/AngII-induced injury. (**A**) Schematic of UniNx/AngII injury and recombination with doxycycline-containing diet. (**B** and **C**) H&E of renal cortices after UniNx/AngII injury and quantification of tubular injury. (**D**) Gene expression of KIM-1 (Havcr1) from injured renal cortices, normalized to Gapdh, was measured by qPCR. (**E**) BUN from plasma at the time of sacrifice. *P < 0.05, **P < 0.01 using 2-tailed Student’s *t* test. Scale bar: 50 μm.

Given the protective effect of inhibiting CDK4/6, we selectively deleted cyclin D1, which complexes with CDK4/6 to promote cell cycle progression (Figure 2A), in renal tubules. To do so, we crossed the doxycycline-inducible Pax8-rTTA;tetO-Cre mouse (15) with the Ccnd1^fl/fl^ mouse to generate Ccnd1^fl/fl^;Pax8-rTTA;tetO-Cre (CyclinD1^CKO^) and confirmed recombination after placing the mice on a doxycycline-containing diet (Supplemental Figure 3). Mice were subjected to UniNx/AngII injury and placed on a doxycycline-containing diet 7 days after the operation (Figure 4A). Contrary to expectations, mice with conditional KO of cyclin D1 had greater tubular injury indicated by greater histological tubular injury, significantly higher gene expression of KIM-1 (Havcr1), and decreased renal function measured by BUN (Figure 4, B–E). There were no significant changes in fibrosis measured by either Picrosirius red or Col1a1 gene expression. Others have reported CDK4/6-independent effects of cyclin D1 (e.g., STAT3 suppression, DNA damage repair) that may be protective in injury (16), and there was a trend toward increased STAT3 signaling in the CyclinD1^CKO^ mice (Supplemental Figure 4). Therefore, unlike inhibition of CDK4/6, deletion of cyclin D1 augmented tubular injury in a CKD murine model.

### Human genetic variants in genes CDKN2B and CCND1 significantly associated with changes in kidney function.

Prior data suggest that cell cycle progression is altered in human CKD kidneys, and our preclinical data suggest that blocking G1/S progression at CDK4/6, but not cyclin D1, is protective in chronic kidney injury. To assess whether this translates to human CKD, we examined whether eQTLs for *CCND1* (cyclin D1), *CDKN2B* (p15), and *CDKN2A* (p16) were associated with eGFR in GWAS (Table 1). Of the 31 eQTLs, 4 for *CCND1* and 1 for *CDKN2B* were significantly associated with eGFR in GWAS. For the significant eQTL for *CDKN2B* (rs9632884), lower gene expression correlated with lower eGFR, consistent with our preclinical data in which greater blockade of CDK4/6, the target of palbociclib and *CDKN2A/B*, was protective in CKD (Table 1). Conversely, the 4 eQTLs for *CCND1* showed that higher levels of cyclin D1 were associated with higher eGFRs, consistent with the murine conditional KO mice. Thus, human genetic data suggest that reduced expression of CDK4/6, but not cyclin D1, protects against CKD in humans, consistent with our pharmacologic and genetic data in murine kidney injury.

### Inhibiting CDK4/6 protects PT cells in vitro from cell death.

**Figure 5:**
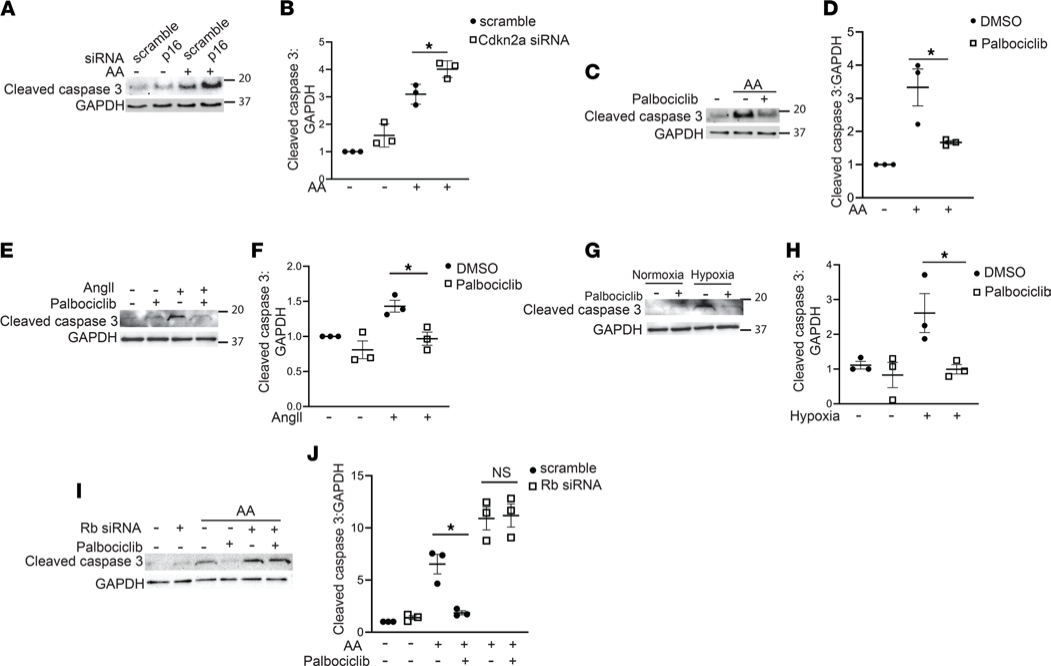
Blocking CDK4/6 reduces tubule cell death. (**A** and **B**) Immunoblots and quantification of cleaved caspase 3 from immortalized PT cells treated with siRNA to Cdnk2a (p16) or scramble and incubated with 30 μM AA for 3 days with GAPDH as a loading control. (**C**–**H**) Primary PT cells were treated with 15 μM AA, angiotensin II 1 μM, or 1% O_2_ (hypoxia) for 7 days plus either palbociclib (2 μM) or equal volume DMSO (diluent) and immunoblotted for cleaved caspase 3 with quantification of 3 separate experiments. (**I** and **J**) Immortalized PT cells were transfected with siRNA to Rb or scramble, treated with 30 μM AA for 3 days, and immunoblotted for cleaved caspase 3 or GAPDH as loading control. *P < 0.05 using the 2-tailed Student’s *t* test. AA, aristolochic acid; Rb, retinoblastoma.

Chronically injured mice treated with palbociclib had fewer TUNEL^+^ tubule cells (Figure 2, O and P), but given the systemic administration of palbociclib, we investigated whether modulation of cell cycle at the G1/S stage has a direct effect on PT cell survival. Conditionally immortalized PT cells were transfected with siRNA to Cdnk2a (p16), which blocks CDK4/6, and effective knockdown was verified (Supplemental Figure 5). Cells with Cdkn2a knocked down had increased cell death, measured by cleaved caspase 3, after 3 days of treatment with AA compared with scramble-treated controls (Figure 5, A and B). Primary cells enriched for PT cells (primary PT cells) were characterized (Supplemental Figure 6) and treated with AA, and cell death was ameliorated by palbociclib compared with DMSO control (Figure 5, C and D). To more closely model the injury induced by UniNx/AngII, primary PT cells were also treated with either AngII or hypoxia (1% O_2_), a common feature of the chronically injured kidney (17). Hypoxia was validated with gene expression of the hypoxia-inducible factor Egln3 (Supplemental Figure 7). Consistent with the AA-treated cells, primary PT cells treated with palbociclib were protected from cell death induced by either AngII or hypoxia (Figure 5, E–H).

We then investigated whether the protective effect of palbociclib depends upon its effects on cell cycle progression. We knocked down Rb, the target of CDK4/6 that leads to cell cycle progression through E2F-dependent transcription (Figure 2A), negating the effects of palbociclib on the cell cycle (Supplemental Figure 8). As expected, palbociclib had a significant protective effect on survival in AA-treated PT cells, but this was lost in cells treated with siRNA-Rb (Figure 5, I and J), suggesting that the protective effect of palbociclib requires the presence of Rb. Taken together, data from genetic and pharmacologic inhibitors show that blocking CDK4/6 promotes PT cell survival in vitro and that this protection is cell cycle dependent.

### Inhibiting CDK4/6 protects PT cells through a STAT3/IL-1β pathway.

**Figure 6:**
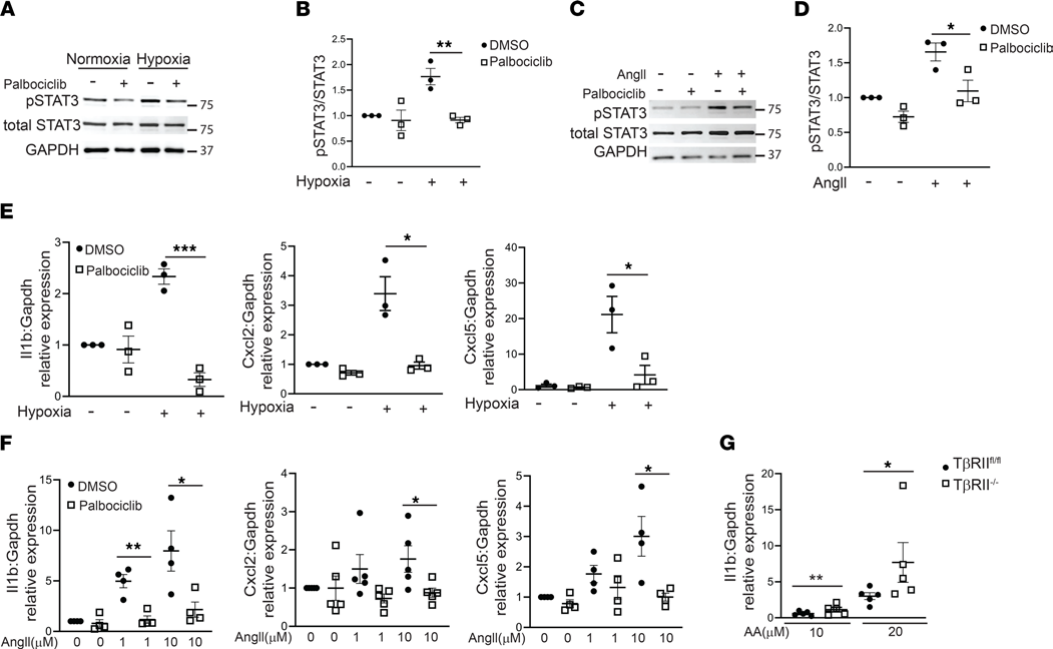
Palbociclib reduces STAT3 activation and downstream targets. (**A**–**D**) Primary PT cells were treated with hypoxia (1% O_2_) or 1 μM angiotensin II (AngII) ± 2 μM palbociclib for 7 days and immunoblotted for phosphorylated STAT3 (pSTAT3), total STAT3, or GAPDH for loading control. (**E** and **F**) Gene expression of IL-1β (Il1b), Cxcl2, and Cxcl5 from primary PT cells treated with hypoxia for 2 days or AngII for 7 days was measured by qPCR with Gapdh as a housekeeping gene. (**G**) Conditionally immortalized proximal tubule cells with and without TβRII were treated with the indicated concentrations of aristolochic acid (AA) for 4 days, and then cDNA was generated for IL-1β (Il1b) gene expression by qPCR. *P < 0.05, **P < 0.01, and ***P < 0.001 using the 2-tailed Student’s *t* test.

Injured PT cells promote tubulointerstitial fibrosis through production of proinflammatory and profibrotic cytokines. Cell proliferation is often linked to the signal transducer and activator of transcription 3 (STAT3) pathway, which has been implicated in proliferation and fibrosis, specifically in kidney diseases (18, 19). The CDK family can directly modulate STAT3, and alterations in STAT3–CDK2/4/6 in human malignancies portend a worse prognosis (20, 21). Thus, we hypothesized that palbociclib may ameliorate tubular injury and tubulointerstitial fibrosis through STAT3 suppression. Primary PT cells treated with hypoxia or AngII had increased STAT3 phosphorylation (i.e., activated STAT3), but this was attenuated by palbociclib treatment (Figure 6, A–D). Others have shown that inhibiting STAT3 in the kidney reduced IL-1β, a proinflammatory cytokine that is also implicated in kidney fibrosis and tubular injury (22, 23). To determine the effect of CDK4/6 inhibition on tubular IL-1β specifically, primary PT cells were treated with hypoxia or AngII, both of which upregulated tubular gene expression of IL-1β (Figure 6, E and F), in addition to CXCL2 and CXCL5, which are cytokines induced by CDK4/6 activity in keratinocytes (24). Furthermore, gene expression levels of these cytokines in primary PT cells treated with hypoxia or AngII were significantly attenuated by palbociclib treatment (Figure 6, E and F). Since TGF-β signaling modulates expression of p15/p16 (Figure 1B), which directly suppress CDK4/6, we examined how deletion of TβRII in conditionally immortalized PT cells affects IL-1β. TβRII^–/–^ PT cells had significantly increased IL-1β gene expression after treatment with AA (Figure 6G). Taken together, our data suggest that blocking CDK4/6 reduces STAT3 activation and IL-1β expression.

**Figure 7:**
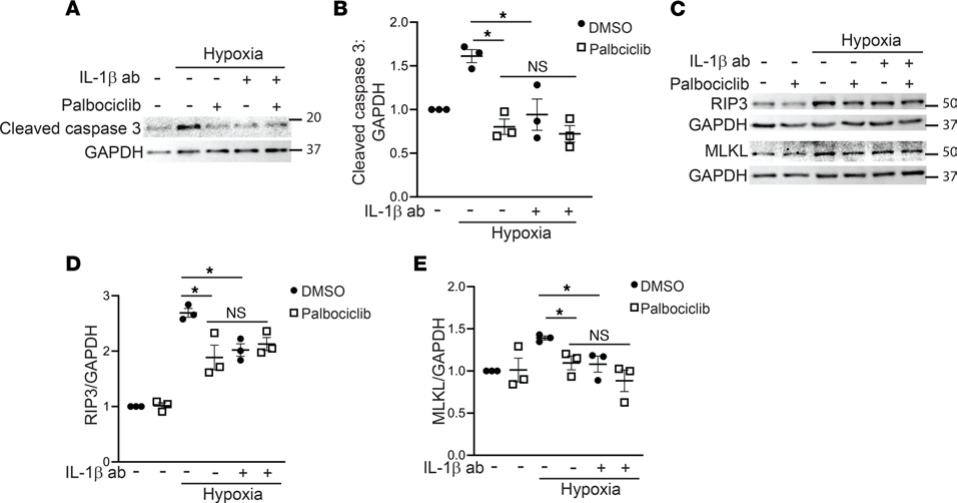
Palbociclib reduces tubular apoptosis and necroptosis, in part, through IL-1β. (**A**–**E**) Primary PT cells were treated with 1% O_2_ (hypoxia) for 7 days plus IL-1β blocking antibody (100 ng/mL), palbociclib (2 μM), or both, and lysates blotted for cleaved caspase 3 (**A** and **B**), or RIP3 and MLKL (necroptosis) and quantified with GAPDH as a loading control (**C**–**E**). *P < 0.05 using ordinary 1-way ANOVA with GraphPad Prism. RIP3, receptor interacting-protein 3; MLKL, mixed lineage kinase domain-like protein.

In prior studies, inhibiting IL-1β reduced tubular injury in human kidney organoids (23), so we tested whether the protective effect of CDK4/6 inhibition in hypoxic primary PT cells could be mediated by IL-1β blockade using a neutralizing antibody to IL-1β. Apoptosis, measured by cleaved caspase 3, was reduced by the IL-1β antibody to a similar extent as palbociclib administration (Figure 7, A and B). Furthermore, there was no additional protection afforded by treating PT cells with both IL-1β antibody and palbociclib, suggesting that the 2 were acting on the same pathway. Cleaved caspase 3 measures apoptosis specifically, and it is debatable as to whether apoptosis is beneficial or detrimental in the context of CKD. Necroptosis, or programmed necrosis, has recently been implicated in renal tubular injury in AKI and CKD (25–27). Necroptosis is induced in cells with high levels of receptor interacting-protein 3 (RIP3), which lead to recruitment of mixed lineage kinase domain-like protein (MLKL) and plasma membrane disruption (28). Hypoxia increased RIP3 and MLKL expression, which were reduced by both palbociclib and IL-1β, but not in an additive fashion (Figure 7, C–E). Thus, blocking CDK4/6 reduces STAT3/IL-1β, and IL-1β is, in part, responsible for the CDK4/6-dependent tubular apoptosis/necroptosis in PT cells after injury.

**Figure 8:**
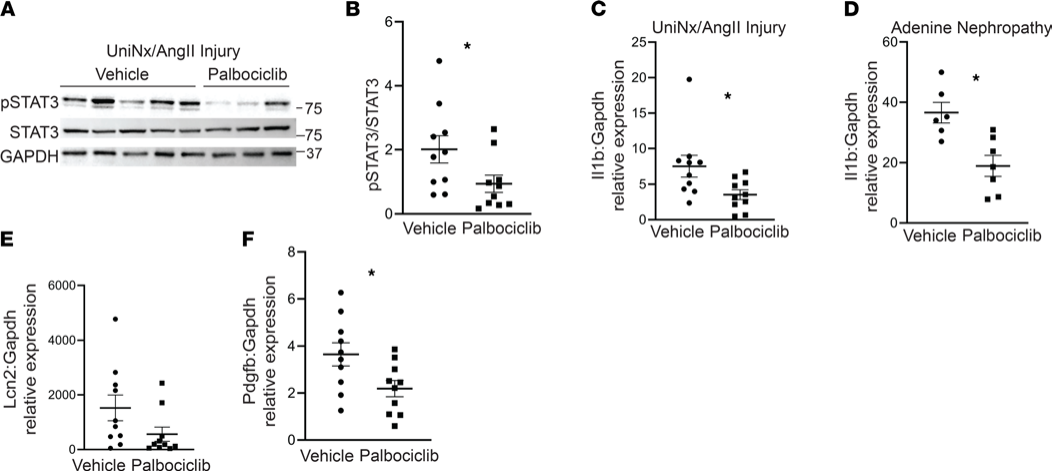
Palbociclib reduces STAT3 activation and IL-1β expression in CKD injury models. (**A** and **B**) Cortical tissue from mice injured by UniNx/AngII was immunoblotted for pSTAT3, total STAT3, and GAPDH for loading control and quantified. (**C** and **D**) Gene expression for IL-1β (Il1b) was measured in renal cortices of UniNx/AngII-injured and adenine nephropathy-treated mice with qPCR using Gapdh as a housekeeping gene. (**E** and **F**) NGAL (Lcn2) and PDGF-β (Pdgfb) were measured in renal cortices of UniNx/AngII injured mice. *P < 0.05 using the 2-tailed Student’s *t* test. NGAL, neutrophil gelatinase-associated lipocalin; PDGF-β, platelet-derived growth factor β.

We then determined whether the protective effects of blocking CDK4/6 on the STAT3/IL-1β pathway observed in PT cells were present in CKD models. Consistent with the in vitro data, palbociclib significantly reduced STAT3 activity in UniNx/AngII-injured mouse cortices compared with vehicle control (Figure 8, A and B). Likewise, gene expression of IL-1β was significantly suppressed in palbociclib-treated kidneys from both UniNx/AngII and adenine nephropathy CKD models (Figure 8, C and D). STAT3 has numerous downstream targets, and other investigators have identified Lcn2, Pdgfb, and Timp1 as profibrotic factors directly downstream of tubular STAT3 activity in the kidney (29). Palbociclib-treated mice injured by UniNx/AngII did have significantly lower levels of Pdgfb and a trend toward reduced Lcn2 (Figure 8, E and F), suggesting that IL-1β is unlikely to be the only mediator of injury downstream of STAT3. In conclusion, blocking CDK4/6 reduces STAT3 activation and IL-1β expression in CKD models and mediates, in part, the protective effects on PT cells in vitro.

## Discussion

Cell cycle progression and proliferation have widely been considered protective in the context of kidney injury and repair. Counter to this paradigm, we show that slowing cell cycle progression at G1/S by blocking CDK4/6 protected against tubule injury, renal functional decline, and tubulointerstitial fibrosis in 2 models of CKD. Palbociclib promoted PT cell survival when cells were subjected to hypoxia, AngII, or AA. The protective effect of palbociclib was dependent upon its actions on the cell cycle, as Rb knockdown abrogated the protective effect. In addition, the STAT3/IL-1β pathway appears to mediate, in part, palbociclib’s protective effect on renal tubules. Importantly, a genetic polymorphism that lowers expression of *CDKN2B*, which also targets CDK4/6, is significantly associated with lower eGFR, suggesting a causal relationship between increased CDK4/6 activity and human CKD.

Few studies have directly investigated the role of cell cycle progression in kidney injury, and nearly all of these prior studies have studied its role in AKI rather than CKD. CKD4/6 inhibitors have been shown to prevent AKI when given prior to either ischemia reperfusion injury (IRI) or cisplatin (30, 31). Induction of cellular quiescence with palbociclib prior to injury likely protects against DNA damage and cell death, as has been shown in models of radiation-induced myelosuppression (32). After AKI, proliferation is critical to reestablish healthy tubules (33), and genetic deletion of Cdkn2a (i.e., p16^INK4a^) improved the tubular atrophy and fibrosis after IRI, likely due to augmented tubular proliferation and reduced senescence (34). Consistent with this, we also treated mice with palbociclib after IRI (begun 4 or 10 days after the ischemic event), but blocking CDK4/6 caused greater mortality and no protective effect at 28 days after injury (data not shown). Thus, blocking CDK4/6 and cell cycle progression is protective in gradually progressive models of CKD (e.g., hypertension) but not after an acute ischemic event when rapid cell cycle progression is critical for repair.

The role of cell cycle progression in CKD, during which time the insult is continuous and indolent, has not been well investigated. We chose the UniNx/AngII model because it is a gradually progressive model of hypertension-induced injury, and hypertension is the second leading cause of end stage kidney disease (ESKD). The beneficial effects of CDK4/6 inhibition were recapitulated in the adenine nephropathy model, another gradually progressive model that targets the PT. Importantly, in both models, administration of palbociclib was started at least 14 days after injury initiation. Counter to reports in the cancer field, palbociclib did not induce senescence, as measured by β-galactosidase staining and p21 levels, in our tissue. Even in the cancer literature, the senescence induced by palbociclib is incomplete, as palbociclib’s effects on the cell cycle are reversible, whereas senescence is an irreversible cell cycle arrested state (35). One feature of senescent cells is that they are growth arrested, despite the presence of mitotic stimuli, and many cancer lines require overactivation of other growth pathways (e.g., MAPK, mTORC1) in order to produce senescence (36, 37). Thus, the ability of palbociclib to induce senescence in noncancerous tissues that lack mutations in these mitogenic pathways is unclear and not supported by our data. Alternatively, more prolonged treatment (>3 weeks) with a CDK4/6 inhibitor may generate different effects on senescence.

Our studies are consistent with prior evidence in the literature suggesting that ongoing tubular cell cycle progression may be detrimental. Epidermal growth factor (EGF) is upregulated after kidney injury and is an important promoter of tubular proliferation. Studies support a protective role for EGF in tubular repair after AKI, but they support a detrimental effect of EGF in CKD (38, 39), raising the question of whether EGF’s profibrotic effects in chronic injury are mediated by ongoing cell cycle progression. Interestingly, several proteins that suppress tubular cell cycle progression (ZAG, HCaRG, and GCM1) have been shown to reduce fibrosis following injury (40–42). However, all of the proteins investigated in these prior studies mediate many cell cycle–independent effects and did not directly assess the role of cell cycle progression in CKD.

A key question is how CDK4/6 inhibition exerts its protective effects. While Rb is the target of CDK4/6 that controls cell cycle progression from G1 to S, CDK4/6 does have other Rb-independent effects (43). However, the protective effect of palbociclib on proximal tubular survival was abrogated without Rb present, indicating that its effects on cell cycle are critical to the protective effects on tubule epithelia. This finding differs from a study in which CDK4/6 inhibition was protective in a psoriasis model but independent from its effects on cell cycle (24). Though palbociclib’s effects on cell survival were dependent upon Rb, we cannot rule out kinase-independent effects, as well. Slowing cell cycle progression at G1/S might be protective for several reasons. By reducing the number of cells that progress to S phase, CDK4/6 inhibition decreases the number of cells that then continue to G2/M arrest, associated with a more profibrotic phenotype (8). Consistent with this, our mice lacking TβRII had increased tubule-specific G2/M arrest after injury (Figure 1, C and D), and TβRII^–/–^ PT cells had expression of Cdkn2a/b, inhibitors of CDK4/6, as well as increased G2/M arrest (5). Additionally, cell cycle progression is energetically demanding, which may not be well tolerated by tubule cells in the hypoxic, chronically injured kidneys. Inducing a quiescent state in injured tubules may lessen the metabolic demands, though further studies are needed to clarify the effect of reduced cell cycle progression on tubular metabolism.

Our data suggest that reducing cell cycle progression through CDK4/6 blockade is protective, in part, through STAT3/IL-1β inhibition. Others have shown that STAT3 is activated in injured kidney tubules and is a well-described mediator of tubulointerstitial fibrosis (18, 44). Proliferating epithelia commonly have increased STAT3 activity, and STAT3 activity is integral to the pathophysiology of podocyte and tubular injury in HIV-associated nephropathy (45). How CDK4/6 specifically activate STAT3 is unclear, but others have shown that CDK4/6 promote STAT3 activation through the methyltransferase EZH2 or directly by binding to the STAT3 promoter (20, 24). Recent data link activity of several CDKs with proinflammatory cytokine production (20), raising the broader question of whether proliferating epithelia may generate an inflammatory microenvironment. STAT3 induces many downstream cytokines, including IL-1β, and our in vitro data suggest that palbociclib may be protective through its blockade. IL-1β can be injurious to PT cells and activate stromal cells to myofibroblasts (23). However, the antifibrotic effects of palbociclib may be mediated by other factors downstream of STAT3 such as PDGF-β, the expression of which was also reduced by palbociclib in UniNx/AngII-injured mice (Figure 5H).

To genetically target cell cycle progression in kidney tubules, we generated mice with tubule-specific deletion of cyclin D1, which complexes with CDK4/6 to phosphorylate Rb. Surprisingly, CyclinD1^CKO^ mice sustained greater tubular injury when recombination was induced after initiation of a hypertensive model of injury. Cyclin D1 has potentially protective actions independent of CDKs, including activation of estrogen receptor–mediated transcription, repression of STAT3, and repair of DNA damage (16, 46–48). The CyclinD1^CKO^ mice had a nonsignificant trend toward increased STAT3 expression and activation (Supplemental Figure 4), and the exact mechanism of exacerbated tubular injury is beyond the scope of this study. However, the discrepant results between the palbociclib-treated and CyclinD1^CKO^ mice is likely due to CDK-independent effects of cyclin D1.

The beneficial effects of blocking CDK4/6 are translatable to human CKD, as diminished expression of *CDKN2B* significantly correlated with decreased renal function (lower eGFR) in patients. Others have described that the *CDKN2B-AS1* (antisense 1) variant is associated with coronary artery disease and type 2 diabetes (49–51), and variants in *CCND1* have been associated in GWAS with type 2 diabetes, hemoglobin A1c, and BMI (51–53). However, to our knowledge, no prior studies have shown *CDKN2B* or *CCND1* eQTLs associating with kidney disease, as described in this manuscript.

In summary, our data challenge the assumption that renal tubular cell cycle progression is always beneficial in the context of renal injury. Reducing cell cycle progression using an FDA-approved selective CDK4/6 inhibitor protected against tubular injury and fibrosis after the initiation of 2 CKD injury models. Pharmacologic and genetic manipulation of cell cycle regulators in CKD preclinical models, as well as human genetic data, suggest that reducing CDK4/6 activity, but not cyclin D1, may be a novel therapeutic strategy for treating CKD.

## Methods

### Animal models.

We purchased FVB and Balb/c mice from the Jackson Laboratory and Charles River Laboratories, respectively, and γGT-Cre;Tgfbr2^fl/fl^ mice were generated on N10 of FVB background as previously published and characterized (9). Mice were crossed onto the mT/mG reporter mouse (54) (backcrossed to N10 FVB) or R26Fucci2aR (Fucci, mixed background) cell cycle mouse from Ian Jackson (The University of Edinburgh, Edinburgh, United Kingdom) (11). Cyclin D1 was selectively and inducibly deleted in renal tubules by crossing the Pax8-rtTA mice with the tetO-Cre (15) and the Ccnd1^fl/fl^ mice (gift from Peter Sicinski, Harvard University, Cambridge, Massachusetts, USA). For all injury experiments, we used male mice 8–12 weeks old. For AA nephropathy, mice were injected with 4 mg/kg of AA (Sigma-Aldrich, A9451) by i.p. injection 6 times over 2 weeks and euthanized 1 or 4 weeks after the last injection. For the UniNx/AngII model, the right kidney was removed and a s.c. osmotic minipump (Alzet, AP-2004) containing AngII delivered at 1.4 mg/kg per day for 4 weeks was placed. For adenine nephropathy model, Balb/c mice were fed 0.25% adenine-containing chow (Sigma-Aldrich, A8626), alternating with regular chow for 4 weeks. Some mice injured by either UniNx/AngII or adenine nephropathy were gavaged (6 days a week) with 150 mg/kg of palbociclib (Pfizer compound-only grant) or vehicle control (0.05N sodium lactate buffer, pH 4). Blood pressure was measured by tail plethysmography (BP-2000 blood pressure analysis system, Visitec systems) in the UniNx/AngII-treated mice.

### Flow cytometry.

We used a previously published protocol (10) where kidney tissues were fixed with 4% paraformaldehyde and protease inhibitors for 30 minutes prior to permeabilization; they were then incubated with collagenase type I (Thermo Fisher Scientific) and dispase II (Invitrogen) for 1 hour. Samples were passed through a 16.5 gauge needle; then, they were passed through a 20 gauge needle and a 50 μM filter. After centrifugation at 5000*g* for 5 minutes at 4°C, samples were incubated with Fc blocking antibody (CD16/CD32 rat anti-mouse, clone 2.4G2, BD Biosciences) followed by anti-GFP (Novus Biologicals, catalog NB600-308), anti–rabbit Alexa Fluor 488 (Cell Signaling Technology, catalog 4412S), and DAPI (Cell Signaling Technology). Data were acquired with BD LSR Fortessa Analyzer at the VUMC Flow Cytometry Shared Resource and analyzed by FlowJo (Becton Dickinson).

### Tubule injury score.

Tubular injury was scored by pathology colleagues who were blinded to the treatment status of the mice. All nonoverlapping fields in cortex were scored (400× magnification) using the following tubular injury system: 0 = no injury; 1 = 1%–25%; 2 = 26%–50%; 3 = 51%–75%; 4 = 76%–100%. Tubular injury was defined as protein cast formation, tubular dilatation, tubular cell swelling, vacuolization, or tubular degeneration.

### IHC and staining.

Paraffin-embedded tissues were rehydrated, and antigen target retrieval was performed with 100 mM Tris buffer (pH 10) in a pressure cooker. For the collagen I, the slides were incubated with collagenase I (Sigma-Aldrich, C0130). The following primary antibodies were used: GFP (Novus Biologicals, NB600-308), Ki-67 (Abcam, ab15580), phospho–histone H3 (Santa Cruz Biotechnology Inc., SC-8656-R), F4/80 (Abcam, ab6640), and collagen I (Abcam, ab34710). Tissues were incubated with the appropriate biotinylated secondary antibody (Vector Laboratories), amplified with ABC Elite peroxidase (Vector Laboratories), and detected by DAB (Millipore Sigma, D4293). Images were taken with Nikon Eclipse E600 or Nikon Eclipse Ti microscopes. For quantification of staining, 10 fields were taken per sample and quantified using ImageJ (NIH). TUNEL assay was performed using ApopTag Plus Peroxidase in Situ Apoptosis Kit (Sigma-Aldrich, s7101) according to the manufacturer’s instructions. Ten fields (×200) per samples were taken, and TUNEL^+^ cortical tubular cells were counted. Rehydrated paraffin-embedded slides were incubated overnight with Picrosirius red (Sigma-Aldrich, 36-554-8), briefly washed in acetic acid, and dehydrated. For senescence β-galactosidase staining, kidney tissues were incubated in 4% paraformaldehyde for 2 hours and 30% sucrose overnight at 4°C, and they were then embedded in OCT. Frozen tissue sections were stained with the Cell Signaling Technology senescence β-galactosidase staining kit (catalog 9860). Ten fields at 400× were taken of the renal cortex, and β-galactosidase^+^ staining was quantified with ImageJ.

### BUN measurement.

Whole blood was collected in heparinized tubes, then centrifuged at 2000*g* for 5 minutes at 4°C, and BUN on plasma measured with the Thermo Infinity Urea Reagent (Thermo Fisher Scientific, TR12421) except for adenine-induced injury and CyclinD1^CKO^-injured mice, which were measured by the Quanti-Chrom Urea Assay Kit (BioAssay Systems, DIUR-100).

### Quantitative PCR.

For RNA extraction, renal cortices were incubated in Lysis Matrix Tubes (MP Biomedicals) containing RLT lysis buffer (Qiagen) before clarification using RNeasy spin columns. RNA from tissue and cells was extracted using the Qiagen RNeasy Kit, and Bio-Rad’s iScript cDNA Synthesis Kit generated cDNA. Quantitative PCR (qPCR) was performed with 100 ng cDNA and SYBER Green Supermix using the Bio-Rad CFX96 Thermal Cycler or QuantStudio 6Flex rT-PCR Systems (Applied Biosystems). Relative mRNA expressions were determined by ΔΔCT equation with Gapdh as a reference gene.

### Immunoblots and reagents.

Kidney tissue was minced in lysate buffer (150 mM NaCl, 50 mM Tris-HCL, 1 mM EDTA, and 2% SDS) plus phosphatase and protease inhibitors, sonicated, centrifuged at 18,800*g* for 10 minutes at 4°C, and reduced with dithiothreitol (DTT) (manufacturer information in Supplemental Table 1). Cells were lysed using MilliporeSigma’s Mammalian Lysis Buffer plus protease and phosphatase inhibitors, sheared using an insulin syringe, clarified by centrifugation at 18,800*g* for 10 minutes at 4°C, and quantified using the BCA protein assay (Thermo Fisher Scientific). Both cell and tissue proteins were separated by SDS-PAGE, transferred to nitrocellulose membranes, and incubated with the following primary antibodies from Cell Signaling Technology: cleaved caspase 3 (9664S, clone 5A1E), pSTAT3 (9145S, clone D3A7), total STAT3 (12640S, clone D3Z2G), α-tubulin (DM1A, clone 3873S),MLKL (37705S, clone D6W1K), pRb (9308, Ser807, clone 811), and cyclinD1 (55506T, clone E3P5S). Additionally, the following antibodies were used: GAPDH (sc-25778, clone K0615, Santa Cruz Biotechnology Inc.); collagen I (AB765P, MilliporeSigma); RIP3 (2283, Pro Sci); CDKN2A/p16^INK4a^ (ab211542, Abcam). Appropriate HRP-conjugated secondaries were used followed by ECL, and bands were detected by GeneGnome (Syngene) or ChemiDoc MP (Bio-Rad); they were quantified by ImageJ.

### RNA-Seq.

RNA-Seq was performed on IMCD cells with and without TβRII as previously described and published (5). Benjamini-Hochberg correction for multiple testing was performed to determine significance, and the data have been deposited in gene expression omnibus (GEO; https://www.ncbi.nlm.nih.gov/; accession no. GSE188317).

### Cells and in vitro experiments.

Conditionally immortalized PT cells with and without TβRII were generated and characterized as previously described (9). PT cells were grown at 33°C in DMEM/F12 supplemented with 2.5% FBS, and PT supplements (hydrocortisone, insulin/transferrin/selenium, triiodothyronine) and penicillin/streptomycin were supplemented with IFN-γ. Before experiments, PT cells were moved to 37°C, and IFN-γ was removed. The PT-enriched primary cells (primary PT cells) were generated as previously described (55) and incubated using RPMI 1640 containing 5% FBS, PT supplements, and penicillin/streptomycin. To induce hypoxia, primary PT cells were exposed to 1% O_2_ in an Invivo2 200 hypoxia chamber (Ruskinn Technologies) for 2, 3, or 7 days. Primary PT cells were also treated with AngII daily (MilliporeSigma, A6402) or AA (MilliporeSigma, A9451) for 7 days. Some cells were treated with palbociclib (Selleck Chemicals, S1116), IL-1β antibody (R&D, AF-401-SP), or both. For siRNA knockdown studies, immortalized PT cells were transfected with nontargeting siRNA (Thermo Fisher Scientific, 4390843), Cdkn2a/p16 targeting siRNA (Thermo Fisher Scientific, 4390771), or Rb targeting siRNA (Dharmacon, L-047474-00-0005) (see Supplemental Methods for sequences) using Lipofectamine RNAiMAX (Thermo Fisher Scientific, 13778-150).

### GWAS and human eGFR.

The set of 31 independent eQTLs selected for evaluation included those within 1 MB of gene transcription start sites (cis-eQTLs), which were significantly associated with proximal gene expression of *CCND1* (cyclin D1), *CDKN2B* (p15), or *CDKN2A* (p16) (*P* < 5 × 10^–6^) in 44 human tissues from the Genotype-Tissue Expression (GTEx) V8 data release. Genome-wide associations with eGFR were obtained from CKDGen summary statistics (56). We used a Bonferroni-corrected *P* value of 0.05/31 = 1.6 × 10^–3^ to declare a significant association between the eQTL and eGFR.

### Statistics.

We used GraphPad Prism to perform all statistics (except for RNA-Seq data), and the 2-tailed Student’s *t* test was used to compare 2 sets of data, with *P* < 0.05 considered statistically significant. For multiple comparisons, the ordinary 1-way ANOVA was used. Experiments subject to analysis were performed at least 3 times.

### Study approval.

All procedures were approved by the IACUC of VUMC and conducted according to the *Guide for the Care and Use of Laboratory Animals* (National Academies Press, 2011).

## Author contributions

YO performed most of the experiments and helped draft the manuscript. MM and AVI both performed some of the in vivo and flow cytometry studies. MPM helped with the mouse genotyping and experiments. JK assisted with the experiments and figure generation. JC helped specifically with the UniNx/AngII model. HY and ABF helped with the injury quantification and manuscript editing. MZ helped with the animal gavage and blood pressure measurement. CRC and NV conducted the analyses with GWAS and human tissues, produced Table 1, and assisted in writing/editing the manuscript. LSG designed and oversaw the experiments and helped in the writing and editing of the manuscript.

## Supplementary Material

Supplemental data

## Figures and Tables

**Table 1:**
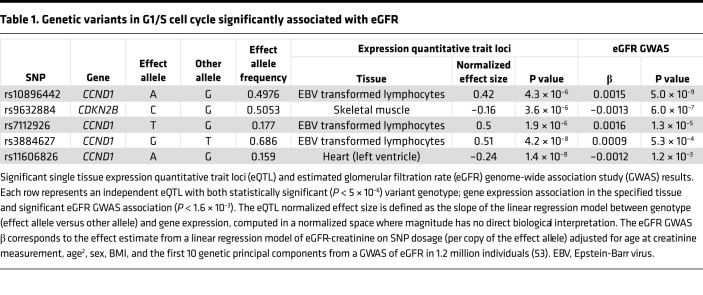
Genetic variants in G1/S cell cycle significantly associated with eGFR

## Version 1 06/22/2022

Electronic publication
