# Supplementary material for: Blocking cell cycle progression through CDK4/6 protects against chronic kidney disease
Source: JCI Insight. 2022 Jun 22;7(12):e158754. doi: 10.1172/jci.insight.158754 (PMC9309053; doi:10.1172/jci.insight.158754)
Supplement: Supplemental data [file jciinsight-7-158754-s069.pdf]

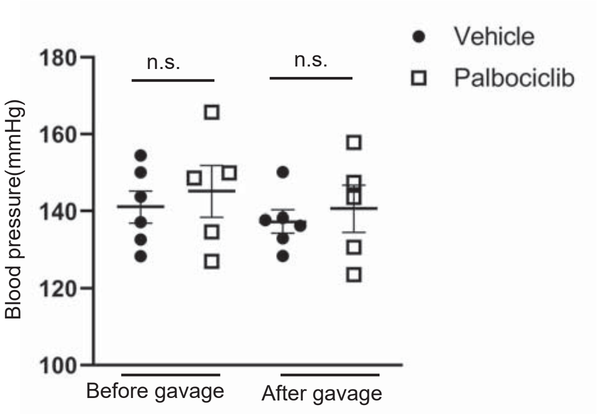

Supplemental Figure 1. Blood pressure was measured by tail pressure plethysmography after UniNx/AngII injury initiated, both before (days 12-17) and after gavage of palbociclib or vehicle (days 21-26). Palbociclib had no independent effect on blood pressure.

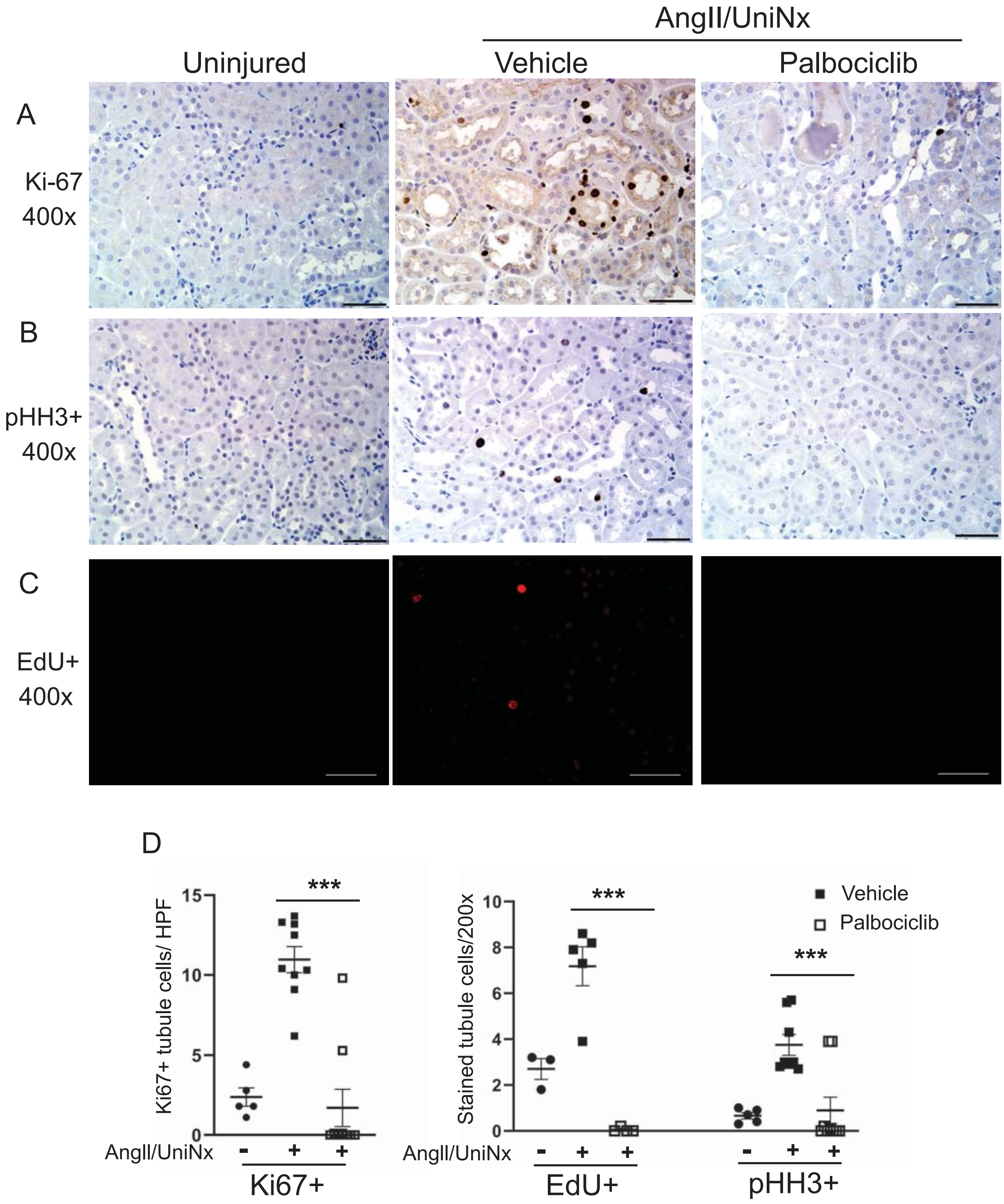

Supplemental Figure 2. Kidney tissue either uninjured or treated by uninephrectomy/angiotensin II (UniNx/AngII) +/- palbociclib treatment was immunostained for Ki-67 (A), phospho-histone H3 (pHH3) (B), or EdU (C) and quantified.

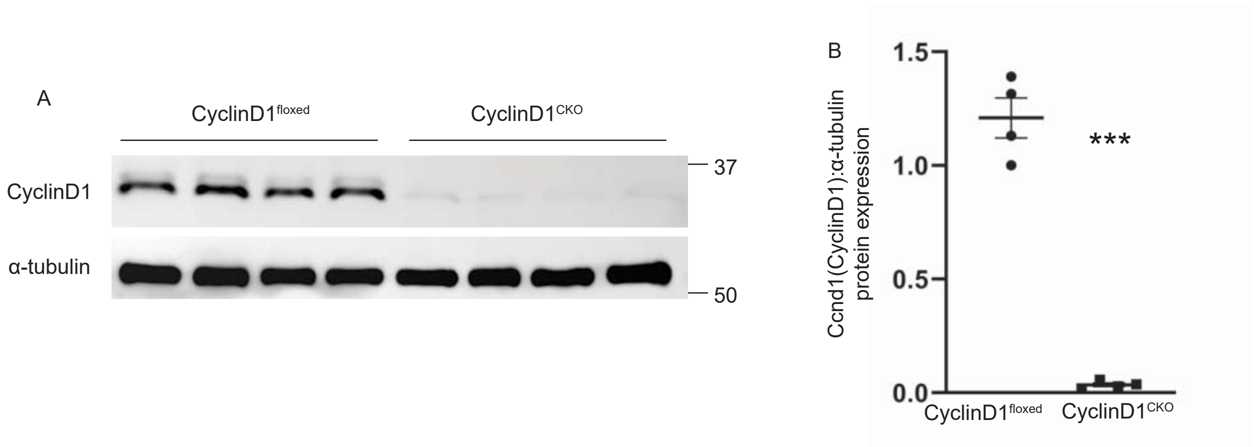

Supplemental Figure 3. CyclinD1^{floxed} (Ccnd1$^{fl/fl}$) and CyclinD1^{CKO} (Ccnd1$^{fl/fl}$;Pax8-rTTA;tetO-Cre) mice were treated with doxycycline-containing diet for 3 weeks, then kidneys were extracted and primary PT cells generated and immunoblotted for cyclin D1 (A) and quantified (B) with α-tubulin as loading control. *** = p<0.001.

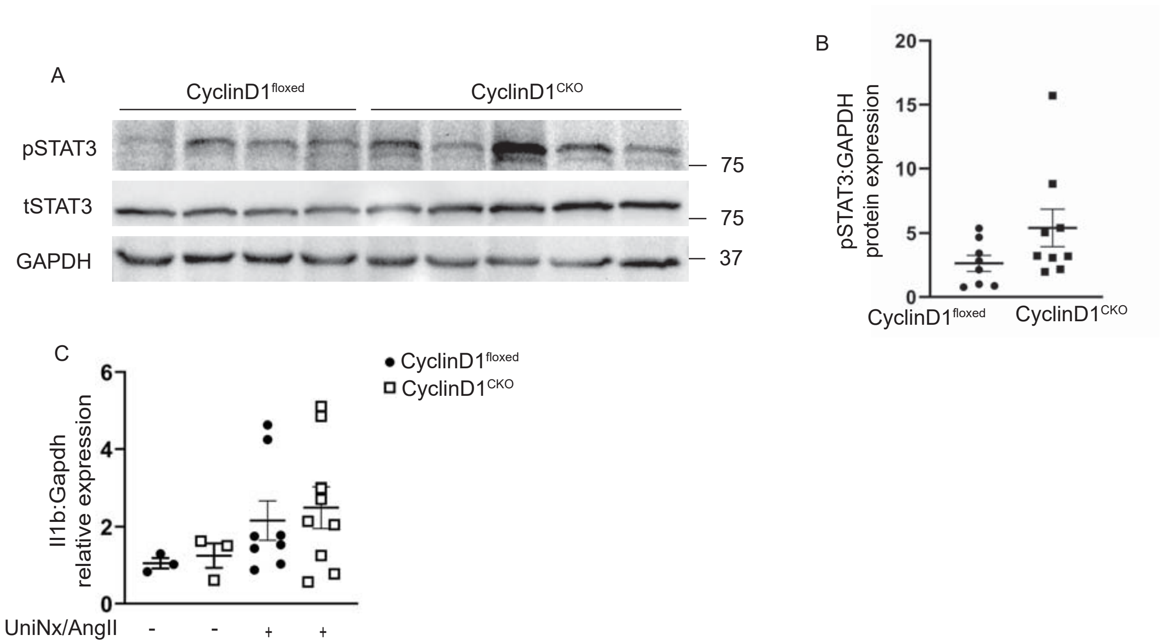

Supplemental Figure 4. Renal cortices from UniNx/AngII-injured CyclinD1^{floxed} (Ccnd1$^{fl/fl}$) and CyclinD1^{CKO} (Ccnd1$^{fl/fl}$;Pax8-rTTA;tetO-Cre) mice were immunoblotted for phosphorylated STAT3 (pSTAT3), total STAT3, and GAPDH (A) and quantified (B). Gene expression of IL-1β (Il1b) was measured in renal cortices using qPCR (C).

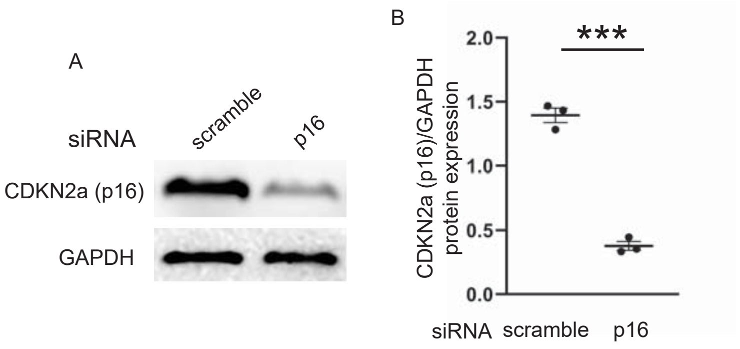

Supplemental Figure 5. Immunoblot of immortalized proximal tubule cells treated with siRNA to either Cdkn2a or scramble (A) and quantification of kncokdown based upon 3 different experiements. 888 = p<0.001.

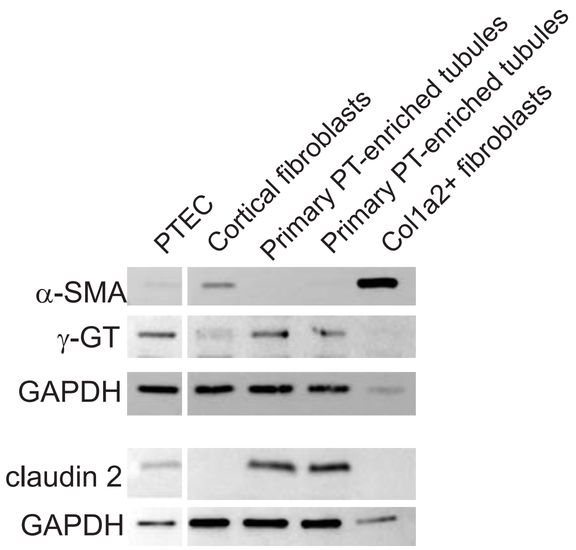

Supplemental Figure 6. Primary PT-enrichied cells ("primary PT cells") were characterized by expression of proximal tubular markers γ-GT and claudin 2 and fibroblast marker α-SMA. Conditionally immortalized PTEC (proximal tubule epithelial cells) and two different fibroblast cell lines were used as controls. GAPDH was used as a loading control and the white line represents lanes that were moved within the same membrane.

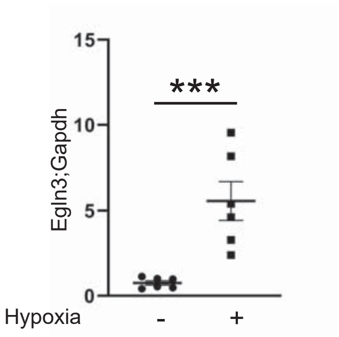

Supplemental Figure 7. Primary PT cells were incubated in hypoxic conditions (1% O_2) for 2 days and expression of Egln3, normalized by Gapdh, was measured by qPCR.
*** = $p<0.001$

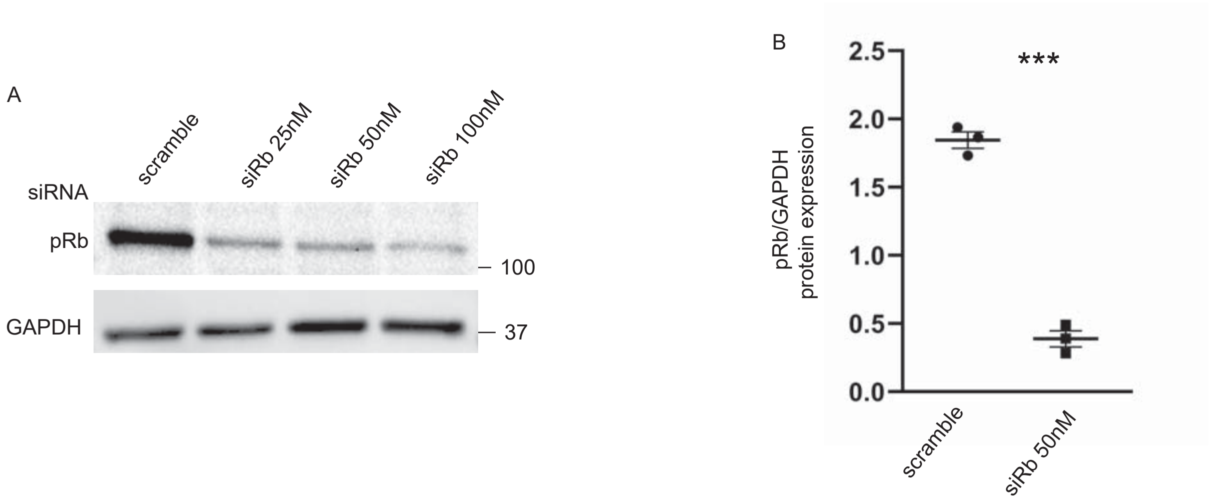

Supplemental Figure 8. Conditionally immortalized PT cells were treated with siRNA to retinoblastoma (Rb) at various concentrations listed (A) and immunoblotted for pRB expression with GAPDH as a loading control. Three independent experiments were done using siRNA to Rb at 50nM and quantified (B). *** = p<0.001.

Supplemental Methods:

Primer sequences for genes used in aPCR are as follows: Primer sequences are as follows (forward and reverse): Gapdh: 5′-AGGTCGGTGTGAACGGATTTG-3′ and 5′-TGTAGACCATGTAGTTGAGGTCA-3′; Havcr1 (KIM-1): 5′-AAACCAGAGATTCCCACACG-3′ and 5′-GTCGTGGGTCTTCCTGTAGC-3′; Col1a1: 5′-GGGTCTAGACATGTTCAGCTTTGTG-3′ and 5′-ACCCTTAGGCCATTGTGTATGC-3′; Cxcl2: 5′- CGCCCAGACAGAAGTCATAGC -3′ and 5′- CTTTGGTTCTTCCGTTGAGGG -3′; Cxcl5: 5′- CCCTACGGTGGAAGTCATAGC -3′ and 5′-GAACACTGGCCGTTCTTTCC -3′; Il1b: 5′- GCCACCTTTTGACAGTGATGAG -3′ and 5′-AAAGGTTTGGAAGCAGCCCT -3′; Nfkbiz: 5′- AACTCGCCAAGAGACCAGTG -3′ and 5′-AGAGCCACTGACTTGGAACG -3′; Cdkn2a (p16) : 5′- CTCTGGCTTTCGTGAACATG -3′ and 5′- TCGAATCTGACCGTAGTTG -3′; Cdkn2b (p15) : 5′- CCTGCCGGTAGACTTAGCTG -3′ and 5′- GAACCCGCAGTCAATCTCCA -3′; Egln3 : 5′- TGCCACCAGGTACGCTATGA -3′ and 5′-TCCTGAATTTCTTTTTGGCTTCTG -3′; Cdkn1b (p27): 5’- AGTGTCCAGGGATGAGGAAG-3’ and 5’- CTTCTGTTCTGTTGGCCCTT-3’; Cdkn1a (p21): 5’- CAAAGTGTGCCGTTGTCTCT-3’ and 5’- TCTCCGTGACGAAGTCAAAG-3’

SiRNA targeted sequences are as follows:
Cdkn2a targeting siRNA (ThermoFisher Scientific 4390771), s201153,sense;5′-GCUGGGUGGUCUUUGUGUAtt -3′ and antisense;5′- UCACAAAGACCACCCAGCgg -3′)
Rb targeting siRNA (Dharmacon, L-047474-00-0005; 5′-GCAUAUCUCCGACUAAAUA -3′, 5′-GGAGGUAACAUCUAUAUAU -3′, 5′-UGCGUUAUCUACUGAAAUA -3′ and 5′-GGACGUGUGAACUUAUAUA -3′)
